# Nomadic souls: Exploring the Mental Health Journey of Migrants through Art

**DOI:** 10.1192/j.eurpsy.2025.402

**Published:** 2025-08-26

**Authors:** S. Regev, V. Gailis, A. Smoliarova

**Affiliations:** 1 Occupational Therapy, Ben Gurion University of The Negev, Be’er Sheva; 2 Vera Gailis - independent art curator, Haifa; 3 Center for Germant Studies, Hebrew University of Jerusalem, Jerusalem, Israel

## Abstract

**Introduction:**

*Nefashot*, meaning “Souls” and “People” in Hebrew, is an initiative dedicated to promoting mental health awareness through cultural and artistic expressions, fostering inclusive dialogue. Art serves as a critical tool in this mission, using its visual language to make difficult conversations possible and amplify voices that are often unheard.

**Objectives:**

The “Nomadic souls” project aims to raise awareness about the intersection of migration and mental health through the presentation of high-quality contemporary art in exhibitions, curated by professional curators and researchers. By showcasing diverse artistic perspectives, it seeks to foster deeper understanding and dialogue around these critical issues.

**Methods:**

This project is a collaboration between *Nefashot*, the art initiative *KAKDELART*, and curators Vera Gailis and Anna Smoliarova, who together launched a call for Israeli artists with diverse immigrant backgrounds under the theme “Nomadic Souls.” The call invited artworks exploring the connection between migration and mental health. So far, the project has produced two exhibitions: “Being Singular/Plural,” which centered on the concept of belonging, and “A Semiotics of the Start,” which delved into the experience of language acquisition. Each exhibition was accompanied by events like literature readings, artist networking, and pop-up photo exhibitions, fostering community engagement and inclusivity.

**Results:**

The project made significant strides in building collaborations and laying the foundation for a platform that facilitated discussions on migration and mental health within both immigrant and local communities. It successfully increased public awareness of these issues and established sustainable partnerships that continued to support the community. Community engagement was central to the project. The exhibitions and related events actively involved migrants as participants, artists, and audience members. Collaborations with the city’s youth department for immigrant assistance were crucial, including co-funding an event that attracted a relevant and engaged audience. Beyond the exhibitions’ objecthood, the project addressed deeper issues such as suicide, anxiety, and depression. Additionally, an academic webinar with immigration professionals was held, focusing on the second exhibition catalog, further broadening the project’s impact and reach.

**Image 1:**

**Figure:**
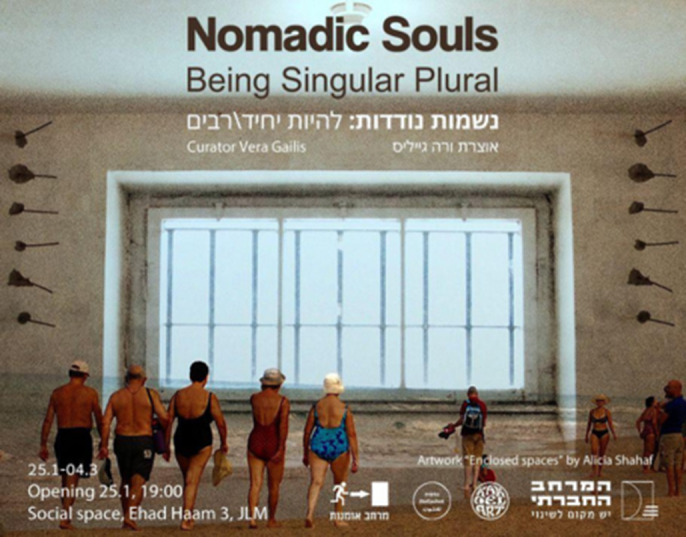

**Conclusions:**

The *Nefashot* initiative highlights the power of art and community collaboration in addressing complex mental health issues related to migration. By fostering dialogue and partnerships, the project has expanded its reach, connecting both local and migrant communities in meaningful ways. The ongoing and future exhibitions aim to continue this momentum, establishing a lasting platform for dialogue, collaboration, and support.

**Disclosure of Interest:**

None Declared

